# Supplementary material for: Towards gender-inclusive innovation: Assessing local conditions for agricultural targeting
Source: PLoS One. 2022 Mar 24;17(3):e0263771. doi: 10.1371/journal.pone.0263771 (PMC8947084; doi:10.1371/journal.pone.0263771)
Supplement: Attachment — Submitted filename: Respose to Reviewers.docx [file pone.0263771.s001.docx]

**Responses to comments from reviewers**

Manuscript ID PONE-D-21-04610

Lopez et al. ‘Towards gender-inclusive innovation: assessing local conditions for agricultural targeting’

**Reviewer #1**

| **Comments from Reviewer** | **Response** |
| --- | --- |
| 1. Thank you for allowing me to read your paper. First, the concept of gender climate to explore how gender norms in specific geographic contexts is insightful, as is the approach to explore this in concert with social, economic and infrastructure conditions. The data are impressive. | Thank you for your positive and constructive feedback. |
| 2. The paper describes the general aims in various parts of the paper. Abstract: We examine the gender climate of 70 villages across 13 countries... Introduction: We explore how a gender norms perspective can be integrated together with the economic and infrastructure conditions ... Discussion: ..we proposed an approach to identify patterns and differences across villages .. we tested this approach using gender-related, demographic, etc. All these aims are in the same ball-mark but yet as a specific research question is missing, it is not easy to really see what is the specific focus and to evaluate whether this study achieves its aims. Currently the relatively broad aims are not helpful in establishing a clear contribution. | The introduction in the revised manuscript has been substantially modified to address these concerns. The introduction now clearly specifies the problem and goals of the article. Discussion and Conclusions have also been amended in consequence. An extract of the introduction follows (lines 73-78):  “…Our objective is twofold. First, to empirically test the relationship between gender normative conditions with economic, infrastructure, and population conditions—all of which have been recognized to influence agricultural innovation processes. And second, to develop a replicable, integrative approach that allows to assess local gender and non-gender conditions to inform the design and targeting of AR4D interventions so that—in line with SDG5— they can become gender-inclusive…” |
| 3. The above also links to the focus on innovation in the paper. This paper is strongly framed to understanding how the context (gender climate and other conditions) conditions innovation.  (a) It is not fully clear why the focus is on 'innovation' (as opposed to entrepreneurial activities or agency of these women).  (b) Another concept introduced is 'interventions' that are understood to be embedded in these diverse contexts. What is the relationship between innovations and interventions here? | The reviewer’s description is correct. We address your questions separately below.  (a) The focus is on agricultural research for development (AR4D) interventions and their targeting strategies which, as explained in the introductory paragraph, “tend to focus on technical-technological issues, such as crop genetic improvement, resource use efficiency, or improved pest and disease management.” Innovation in this technical-technological sense is a core element of AR4D research and its interventions. This is not the case, however, with entrepreneurial activities or women’s agentic capacities as AR4D does not necessarily envisage these as key issues in their work. Although this is slowly changing (also because of the UN SDGs, especially SDG-5 gender equality), the focus remains on innovation.    (b) This is partly answered above. In our article, ‘intervention’ refers to the planned activities of an AR4D project which aim to strengthen or develop the agricultural sector of a particular geographic location by providing technical-technological solutions (i.e., innovations). |
| 4. The study finds that the dimensions identified 'connectivity' and 'opportunity' do not translate into an enabling environment for gender inclusive innovation. Through the qualitative case studies, it becomes apparent that the emancipation of women and formation of women associations to gain power and authority are important. | That’s partly correct. As noted in the abstract, “our findings evidence that favourable economic or infrastructure conditions do not necessarily correlate with favourable gender normative conditions“. Also, based on the insights gained from the two case studies, it appears that even when enabling conditions for agricultural innovation linked to ‘Opportunity’ or ‘Connectivity’ are present in a rural village, these conditions do not necessarily translate into relaxed gender climates that facilitate gender-inclusive innovation. Nonetheless, and again based on insights from the case studies, we argue that the elements encompassed under the three components (‘Gender Climate’, ‘Connectivity’ and ‘Opportunity’) are *complementary* in their contribution for creating an enabling environment for gender-inclusive innovation. We have clarified this in the manuscript (Discussion). |
| 5. Further comments on the concepts:  (a) Gender inequity vs. inequality?  (b) Relaxed vs restrictive gender climate? | (a) Thank you for bringing this to our attention. There is no general agreement on the definition and use of inequity and inequality, in the same way that none exists between gender equality and equity— with many using the terms interchangeably (our case). Many gender experts and organizations nonetheless assert differences between the terms. For example, UNESCO uses **gender equity** to refer to ‘fairness of treatment for men and women according to their respective needs…which may include equal treatment or treatment that is different but which is considered equivalent in terms of rights, benefits, obligations, and opportunities.’ Whereas UN Women defines **gender equality** to state that ‘the rights, responsibilities and opportunities of individuals will not depend on whether they are born male or female.’  To avoid confusion we have harmonized the terminology and, with the exception of a reference to SDG5-gender equality, we solely use the terms linked to the UNESCO definition i.e., equity/equitable/inequity.  (b) We added an explanation about the criteria for identifying a climate as restrictive or relaxed in Materials and Methods. The text (lines 229-237) now reads:  “The criteria for identifying a gender climate as ‘restrictive’ or ‘relaxed’ was likewise informed by these works, specifically those of Petesch et al. which identified freedom of mobility and ability to make decisions as key for agricultural innovation (2018a; 2018b). In this article, a restrictive climate refers to formal and informal norms that restrict women’s mobility, education, decision-making and access to assets and resources. Likewise, it encompasses reported violence against women and lack of leadership positions for women. Conversely, a relaxed climate refers to norms that support women’s education, leadership, decision-making, access to assets and resources, and women’s ability to move freely in the public space. It also suggests no reported violence against women.” |

**Reviewer #2**

| **Comments from Reviewer** | **Response** |
| --- | --- |
| 1. This research article sets out to develop a set of indicators of locally prevailing gender norms relevant for agricultural innovations using a systematic replicable approach based on qualitative data collected in a standardized way. It additionally has the objective to empirically demonstrate the relevance of incorporating a gender norms’ perspectives into agri-food systems research. The study uses a rich and high quality qualitative dataset incorporating multiple rural villages in 13 low and middle income countries. The principle component analysis (PCA) clearly identifying three distinct components linked to connectivity, opportunity and gender equity/women’s empowerment is an interesting finding, even if somewhat driven by the available data. | Thank you for the careful consideration of our manuscript. |
| 2. I have several concerns about the method, however. My major concern is that the methods for analysis are not fit to address the research questions. In my view, the study did not empirically demonstrate the relevance of incorporating a gender norms’ perspectives into agri-food systems research. | We apologize for the lack of clarity regarding the identification of the problem and the study objectives. Reviewer #1 has also voiced similar concerns. The introduction has been substantially modified to address this issue. Abstract, Discussion and Conclusions have also been amended in consequence. An extract of the introduction follows (lines 73-78):  “…Our objective is twofold. First, to empirically test the relationship between gender normative conditions with economic, infrastructure, and population conditions—all of which have been recognized to influence agricultural innovation processes. And second, to develop a replicable, integrative approach that allows to assess local gender and non-gender conditions to inform the design and targeting of AR4D interventions so that—in line with SDG5— they can become gender-inclusive…” |
| 3. Furthermore, I am not convinced that the analysis empirically demonstrates that men and women do not necessarily equally benefit from investments in enabling elements for agricultural innovation identified under ‘Opportunity’ (e.g., agricultural and non-agricultural trainings or farmer organizations) and ‘Connectivity’ (e.g., closeness to markets or access to public services). | We apologize for the confusion about the goal of our study but it was not our ambition to empirically demonstrate whether men and women equally benefit from investments in enabling elements for agricultural innovation such as those encompassed under ‘Opportunity’ and ‘Connectivity’. Instead, our goal was to develop an approach that would allow to assess whether gender normative conditions linked to agricultural innovation (encompassed under the ‘Gender Climate’ component) are related to economic, infrastructure, or population conditions encompassed under ‘Opportunity’ and ‘Connectivity’). Hence, our methodology based on two separate datasets and multivariate analyses is useful to demonstrate the complementarity of gender norms and other (non-gendered) village characteristics such as those under ‘Opportunity’ and ‘Connectivity’. We have clarified this throughout the article including in the introduction and discussion. |
| 4. First, because the method of analysis sought orthogonality, not correlation, of aspects of connectivity/opportunity and factors conducive for gender equity, the analysis did not investigate gender equity of connectivity/opportunity. | Again, we apologize for the confusion about the goal of our study. Our purpose was not to investigate the gender equity within connectivity/opportunity. Rather, we used pairwise correlation and variance partitioning analyses to investigate if there existed statistical linkages between the ‘Gender Climate’ component (which according to the cited literature on women’s empowerment and gender norms could enable more gender-inclusive innovation) and the components ‘Opportunity’ and ‘Connectivity’—which, according to the cited literature encompass recognized elements that contribute to the enabling environment for ag innovation, e.g. access to markets, trainings, farmer or agricultural organizations, etc. We have added clarification points in Materials and Methods (see lines 174-176, 202-207, 213-235, 302-312, 335-340, 349-353, and in Table 2). |
| 5. Second, the two Nepalese case studies show that the indicators loading the gender climate component and the presence of gender inclusive innovation may well be endogenous or reversely related (i.e. because there is gender inclusive innovation, women acquired more decision-making power, have more mobility etc). | Indeed, but for this to be proven the selection criteria would have needed to include the presence of gender-inclusive agricultural innovations, which was not the case. To clarify this, and also based on the reviewer’s subsequent comments, we have added more information on the selection criteria. The ‘(b) Sampling’ now reads (lines 202-207):  “GENNOVATE’s selection criteria primarily focused on villages across and within regions with significant heterogeneity in economic, political, agroecological and sociocultural contexts (Petesch et al., 2018c). A secondary criteria considered the presence of AR4D technical-technological solutions in the villages, such as improved seed varieties or agronomic practices, to ensure villagers’ experiences with and exposure to external agricultural interventions (ibid). For a full account on the sampling and methodology see Petesch et al. (2018c).”  Because the technical-technological solutions were introduced to the villages *prior* GENNOVATE and they did not follow comprehensive gender-inclusive principles or strategies, an assessment of the incidence of gender-inclusive innovation is not feasible. |
| 6. Third, apart from the two Nepalese case studies, there is no evidence that either of the three components coincides with gender inclusive innovation. | That is correct, and we were not intending to statistically demonstrate this. As mentioned in response to point 4, the link between the three components and innovation is assumed based on existing literature. The variables used in our analysis are recognized to contribute to an enabling environment for agricultural innovation. |
| 7. In my view, a more convincing analysis of the role of prevailing gender norms for gender inclusive innovation would have consisted of an analysis of correlation between incidence of gender inclusive innovation and measures of opportunity, connectivity, and gender climate/norms. | Indeed, that would have been a very interesting study, however, our data does not allow this kind of analysis because the incidence of gender-inclusive innovation was not measured during the survey. |
| 8. But a challenge would be to find indicators of gender climate/norms that are not likely to have been influenced by (the presence or absence of) gender inclusive innovation. | We agree with this challenge and commented about it in point 5. |
| 9. Another way which could rely on the rich qualitative data could be to look into the incidence of gender inclusive innovation in sets of countries that a) score high on gender climate-high on opportunity-high on connectivity (eg Nepal, Uzbekistan), b) score high on gender climate-low on opportunity-low on connectivity (eg Malawi, Tanzania); c) low on gender climate-high on opportunity-high on connectivity (eg Bangladesh, Pakistan), d) and so on. (And if possible, such analysis by sets of villages with a), b), c) etc could even provide more nuanced insights). | Again, that would be a very interesting study to further confirm our findings. However, because our article does not intend to measure the incidence of gender-inclusive innovation we cannot follow your suggestion.  What our study does, however, is evidence that favourable economic or infrastructure conditions do not necessarily correlate with favourable gender normative conditions. We have amended the introduction, discussion and conclusion to clarify our contribution. An extract of the Discussion follow (lines 576-579:  “Overall, our results indicate that the conditions encompassed under the three components (‘Gender Climate’, ‘Connectivity’ and ‘Opportunity’) are *complementary* in their contribution for creating an enabling environment for gender-inclusive innovation.” |
| 10. Furthermore, although I am not an expert on principle component analysis (PCA), in my view, there are some concerns with the Hill and Smith PCA here. The fact that a pairwise correlation analysis between each of the two complementary components and the gender climate component shows statistically insignificant correlation seems obvious since, in my understanding, PCA seeks orthogonality of the components (i.e. non-correlation). | It is true that PCA seeks orthogonality of components, so ‘Connectivity’ and ‘Opportunity’ are orthogonal and non-correlated by construction. However, we ran two separate PCA-like  analysis (Hill and Smith analysis) on two separate and distinct datasets (the one informing the ‘Gender Climate’ and the one informing the two complementary components of ‘Opportunity’ and ‘Connectivity’). Thus, the fact that ‘Gender Climate’ is not correlated to any of the complementary components ‘Connectivity’ or ‘Opportunity’ is not biased by our methodology. |
| 11. Furthermore, PCA is sensitive to variables’ range and, if unstandardized, variables with larger range tend to dominate. How does the Hill and Smith PCA method take into account diverse ranges of initial (categorical, continuous and dichotomous) variables? The most important variables in the gender climate component seem to be the variables with the widest range, so the way the Hill and Smith PCA method deals with varying ranges needs to be clarified and interference of varying ranges ruled out. | All variables are scaled to remove differences in units and ranges when doing a Hill and Smith analysis. The Hill and Smith method (generalized by Kiers 1994) was developed specifically to consider categorical and continuous variables. We added a clarification in the Materials and Methods section (lines 340-343) and also refer the reader to Hill and Smith seminal work for further details (listed in references):   1. Hill, M. O., and A. J. E. Smith. 1976. Principal component analysis of taxonomic data with multi-state discrete characters. *Taxon*, 25, 249-255. 2. Kiers, H. A. L. 1994. Simple structure in component analysis techniques for mixtures of qualitative and quantitative variables. *Psychometrika*, 56, 197-212. |
| 12. (a) With regard to sampling, it is not clear to me how maximum diversity sampling and an approach to capture central themes that cut across a great deal of variation go together. The latter rather seems an approach to achieve minimum diversity (in the central themes). The authors go on to explain that “the sample sought distribution of villages across and within regions to introduce significant heterogeneity in economic, political, agroecological and sociocultural contexts that underpin gender norms in agricultural innovation”.  (b) This seems to suggest there were prior hypotheses about what economic, political, agro-ecological, socio-cultural characteristics underpin gender norms in agricultural innovation. This raises the question whether there is a risk that by seeking distribution of villages accordingly automatically confirms the hypotheses. | (a) The sampling scheme seeks to capture the maximum diversity within regions. Although unorthodox, such sampling is not incompatible with multivariate analysis which aims to explain the largest amount of variability with the minimum number of components. In fact, maximum diversity sampling ensures that a larger diversity of villages are captured with a minimum number of samples.  (b) As any research article, ours was indeed informed by an interdisciplinary body of literature—including on gender norms and women’s empowerment, agricultural targeting, and enabling environment for agricultural innovation; all of which highlighted important elements that contribute to an enabling environment for both agricultural innovation and gender-inclusive agricultural innovation (such as those under ‘Gender Climate’, ‘Opportunity’ and ‘ Connectivity’). Nonetheless, the village selection criteria was not influenced by these literature searches because our manuscript developed years after the GENNOVATE conceptual framework, methodology and goals were developed. Hence, the authors of this study could not have biased the village selection. |
| 13. Other concerns regarding method include:  (a) Insufficient transparency about sampling, size of sample of villages (e.g. info about the number of villages per country is needed to understand table 3 where a distribution of village scores per country cohort is shown), data from which data collection exercise is used in indicators (FGD ladder of life, capacities for innovation, or youth aspirations, with men or women), tables (with notes) should be self-explanatory (e.g. note on country codes and variable labels).  (b) Furthermore, the section “dimensions and variables” repeats information from table 2 rather than providing a clearer description of the way each of the indicators is measured (based on what questions, answered by whom) and what they measure exactly. | We apologize for the frustration experienced by the reviewer and we have now substantially improved the methodological section.  (a) The revised version of Materials and Methods now expands on the sampling and selection criteria and also refer the reader to a published article that exclusively deals with sampling limitation and strengths. Likewise, we direct the reader to the full GENNOVATE methodology (see Petesch et al., 2018c) –also available on the accompanying tutorial: <https://rfrelat.github.io/GenderClimate.html>  The accompanying Tutorial also provides more information on sampling and variable selection. We have also added the information you rightfully requested for Figure 3 and Tables 2 and 3, and clarified the notes.  (b) As per your suggestion, the description of variables in ‘(c) Dimensions and variables’ are now improved to better reflect how they were collected in the study sites. We also direct the reader to the data collection instruments used, i.e., community profiles and focus group discussions; which are also available as part of the GENNOVATE methodology and in the accompanying Tutorial. Likewise, the Tutorial now includes an Excel file with the questions used to inform the variables. |
| 14. (a) On a conceptual note, I would advise an argumentation why the authors use a concept of gender norms/climate where a set of rules, institutions, customs, norms is only be related to geographical locations. Insights into intersectionality show that, within geographical locations, the set and the extent to which norms/customs/institutions define daily behaviour may vary by population groups that differ by age, wealth, ethnicity, religion, education level etc. | (a) Thank you for bringing this issue forward. Indeed, intersectional analyses that consider different population groups and dynamics--including intergenerational local norms— are necessary for a comprehensive reading of the influence of situated norms and relations for agricultural innovation among distinct types of people in a given location. We added a discussion about this in the manuscript (see lines 636-642). Likewise, in the introduction we now provide references to works that apply an intersectional lens—including some of our own work with intersectionality (e.g. Elias et al., 2018; Farnworth et al., 2018a; Petesch & Badstue, 2019). We also try to highlight the village diversity (albeit superficially) in the two Nepalese case studies. For example, we state that NP1 is a culturally diverse village (including inhabitants from Tharu, Brahmin and Chhetri origins) whereas NP3 is mostly inhabited by a single social group (Chhetri).  Nonetheless, the fine intersectional analysis that you suggest can be better done by using different methods such as in-depth qualitative analyses in a single village, comparative case study analyses of smaller samples, or by quantitatively examining intrahousehold relations and dynamics. The data used for this particular study, however, is not in-depth enough to allow for such rich intersectional analysis. Another important consideration for using a gender rather than an intersectional approach responds to the scale of our analysis. We focused at the level of the village and aimed at comparing 70 villages instead of comparing different households or individuals within a particular village because the focus of our study is the improvement of the targeting strategies of AR4D interventions— many of which use the village as the usual scale for intervention. |
| (b)  And a discussion would be needed to clarify how the concept of gender climate is distinct from (formal, informal) institutions (in the sense of rules of the game)? | (b) Albeit different from other social informal institutions, gender norms are indeed one type of informal institution. We have added a clarification point in the introduction (lines 118-124):  “Gender norms are part of broader, formal and informal, social institutions that regulate people’s interaction in a given community. Unlike other social institutions, however, gender norms are maintained by internalized and stereotypical beliefs about different types of women and men as well as by broader social expectations that individuals should act in gender-appropriate ways (ibid). Due to this, gender norms play a crucial role in shaping men’s and women’s identity, freedom, voice, power, and access to resources (Cislaghi & Heise, 2019).” |
