# Supplementary material for: Towards gender-inclusive innovation: Assessing local conditions for agricultural targeting
Source: PLoS One. 2022 Mar 24;17(3):e0263771. doi: 10.1371/journal.pone.0263771 (PMC8947084; doi:10.1371/journal.pone.0263771)
Supplement: Attachment — Submitted filename: Responses to Reviewers.docx [file pone.0263771.s002.docx]

**Responses to Reviewers**

| **Requirements from Journal** | **Responses from authors** |
| --- | --- |
| 1. Please ensure that your manuscript meets PLOS ONE's style requirements, including those for file naming. The PLOS ONE style templates can be found at  <https://journals.plos.org/plosone/s/file?id=wjVg/PLOSOne_formatting_sample_main_body.pdf> and  <https://journals.plos.org/plosone/s/file?id=ba62/PLOSOne_formatting_sample_title_authors_affiliations.pdf> | We have checked the requirements and made sure all files submitted followed the required style of PLOS ONE. |
| 2. Please review your reference list to ensure that it is complete and correct. If you have cited papers that have been retracted, please include the rationale for doing so in the manuscript text, or remove these references and replace them with relevant current references. Any changes to the reference list should be mentioned in the rebuttal letter that accompanies your revised manuscript. If you need to cite a retracted article, indicate the article’s retracted status in the References list and also include a citation and full reference for the retraction notice. | The reference list has been checked thoroughly. |
| 3. Thank you for stating the following in the Acknowledgments Section of your manuscript:  "The authors acknowledge the financial support of the CGIAR Programs on MAIZE and WHEAT (grant number 2100723600). The views expressed in the article are those of the authors and not of any organization."  We note that you have provided funding information that is not currently declared in your Funding Statement. However, funding information should not appear in the Acknowledgments section or other areas of your manuscript. We will only publish funding information present in the Funding Statement section of the online submission form. Please remove any funding-related text from the manuscript and let us know how you would like to update your Funding Statement. Currently, your Funding Statement reads as follows:  "D.E.L The lead author acknowledges the financial support of the CGIAR Programs on MAIZE and WHEAT (grant number 2100723600). URL for MAIZE: <https://maize.org/>; URL for WHEAT:<https://wheat.org/>  The funders had no role in study design, data collection and analysis, decision to publish, or preparation of the manuscript."  Please include your amended statements within your cover letter; we will change the online submission form on your behalf. | We have removed the acknowledgment of the funder in the manuscript and edited the funding statement.  The amended Funding Statement should read:  "D.E.L The lead author acknowledges the financial support of the CGIAR Programs on MAIZE and WHEAT (grant number 2100723600). URL for MAIZE: <https://maize.org/>; URL for WHEAT:<https://wheat.org/> The funders had no role in the study design, data collection and analysis, decision to publish, or preparation of the manuscript. The views expressed in the article are those of the authors and not of any organization."  We would be grateful if you could change the online submission form to this amended statement on our behalf. |
| 4. Please amend the manuscript submission data (via Edit Submission) to include author Romain Frelat and Lone. B. Badstue. | Thank you, we have amended the manuscript submission data to include the two authors. |
| 5. We note that Figure 1 in your submission contain [map/satellite] images which may be copyrighted. All PLOS content is published under the Creative Commons Attribution License (CC BY 4.0), which means that the manuscript, images, and Supporting Information files will be freely available online, and any third party is permitted to access, download, copy, distribute, and use these materials in any way, even commercially, with proper attribution. For these reasons, we cannot publish previously copyrighted maps or satellite images created using proprietary data, such as Google software (Google Maps, Street View, and Earth). For more information, see our copyright guidelines: <http://journals.plos.org/plosone/s/licenses-and-copyright>.   We require you to either (1) present written permission from the copyright holder to publish these figures specifically under the CC BY 4.0 license, or (2) remove the figures from your submission:   a. You may seek permission from the original copyright holder of Figure 1 to publish the content specifically under the CC BY 4.0 license.     We recommend that you contact the original copyright holder with the Content Permission Form (<http://journals.plos.org/plosone/s/file?id=7c09/content-permission-form.pdf>) and the following text: “I request permission for the open-access journal PLOS ONE to publish XXX under the Creative Commons Attribution License (CCAL) CC BY 4.0 (<http://creativecommons.org/licenses/by/4.0/>). Please be aware that this license allows unrestricted use and distribution, even commercially, by third parties. Please reply and provide explicit written permission to publish XXX under a CC BY license and complete the attached form.”   Please upload the completed Content Permission Form or other proof of granted permissions as an "Other" file with your submission.   In the figure caption of the copyrighted figure, please include the following text: “Reprinted from [ref] under a CC BY license, with permission from [name of publisher], original copyright [original copyright year].”   b. If you are unable to obtain permission from the original copyright holder to publish these figures under the CC BY 4.0 license or if the copyright holder’s requirements are incompatible with the CC BY 4.0 license, please either i) remove the figure or ii) supply a replacement figure that complies with the CC BY 4.0 license. Please check copyright information on all replacement figures and update the figure caption with source information. If applicable, please specify in the figure caption text when a figure is similar but not identical to the original image and is therefore for illustrative purposes only. The following resources for replacing copyrighted map figures may be helpful:   USGS National Map Viewer (public domain): <http://viewer.nationalmap.gov/viewer/> The Gateway to Astronaut Photography of Earth (public domain): <http://eol.jsc.nasa.gov/sseop/clickmap/> Maps at the CIA (public domain): <https://www.cia.gov/library/publications/the-world-factbook/index.html> and <https://www.cia.gov/library/publications/cia-maps-publications/index.html> NASA Earth Observatory (public domain): <http://earthobservatory.nasa.gov/> Landsat: <http://landsat.visibleearth.nasa.gov/> USGS EROS (Earth Resources Observatory and Science (EROS) Center) (public domain): [http://eros.usgs.gov/#](http://eros.usgs.gov/) Natural Earth (public domain): <http://www.naturalearthdata.com/> | Figure 1 was created by the authors using the open source tool R 3.6 (cited in the manuscript lines 347-348). Therefore Figure 1 is not copyrighted and can be published by PLOS ONE under CC BY 4.0. |

| **Comments from Editor and Reviewers** | **Responses from authors** |
| --- | --- |
| **Editor**  1. Dear authors, in order to improve the quality of your manuscript, I suggest you listen to the reviewers' suggestions. And attend the suggested references.  References  1. Zuniga González, C. A., & Villanueva, J. L. J. (2012). Wages and Employs for Non-Farm Agricultural Activities: One Livelihood Strategy in Nicaragua. Global Journal of Management And Business Research, 12(15).  2. Zúniga-González, C. A., Jarquín-Saez, M. R., Martinez-Andrades, E., & Rivas, J. A. (2016). Participative action Research: A Generation Knowledge approach. Ibero-American Journal of Bioeconomy and Climate Change, 2(1), 218–224. | Thank you for the suggestions, interesting works indeed particularly Zúniga-González et al. (2016). However, we did not find specific sections or arguments where these references would strengthen the content of our current manuscript. Therefore, and for sake of clarity, we decided not to add them. |
| **Reviewer #1**  2. Is the manuscript technically sound, and do the data support the conclusions? The manuscript must describe a technically sound piece of scientific research with data that supports the conclusions. Experiments must have been conducted rigorously, with appropriate controls, replication, and sample sizes. The conclusions must be drawn appropriately based on the data presented.  Reviewer #1: No | We are sorry the Reviewer found some of these elements missing. However, the Reviewer’s comment (point 6) do not expand on this or provide suggestions for improvement. On the contrary, the comment overall highlights the scientific contribution of the article. Therefore, and if deemed appropriate, we would appreciate specific feedback on this point. |
| 3. Has the statistical analysis been performed appropriately and rigorously?  Reviewer #1: Yes | Thank you. |
| 4. Have the authors made all data underlying the findings in their manuscript fully available? The PLOS Data policy requires authors to make all data underlying the findings described in their manuscript fully available without restriction, with rare exception (please refer to the Data Availability Statement in the manuscript PDF file). The data should be provided as part of the manuscript or its supporting information, or deposited to a public repository. For example, in addition to summary statistics, the data points behind means, medians and variance measures should be available. If there are restrictions on publicly sharing data—e.g. participant privacy or use of data from a third party—those must be specified.  Reviewer #1: No | Both the dataset as well as a tutorial are available in the public repository GitHub and can be accessed online at https://rfrelat.github.io/GenderClimate.html  (DOI 10.5281/zenodo.4395534)  The manuscript also directs the reader to the public repository (see line 351 in ‘Data analysis’). Likewise, as stated in lines 162-163, the methodology and data collection instruments used in the study are open access and are available at [www.gennovate.org](http://www.gennovate.org) |
| 5. Is the manuscript presented in an intelligible fashion and written in standard English? PLOS ONE does not copyedit accepted manuscripts, so the language in submitted articles must be clear, correct, and unambiguous. Any typographical or grammatical errors should be corrected at revision, so please note any specific errors here.  Reviewer #1: Yes | Thank you. |
| 6. Review Comments to the Author.  The scientific evaluation of the article with the gender perspective has a global impact, it will serve as the basis for other investigations because it has a relational character with the dimensions and variables which involve cultural, economic, political aspects, among others. Next, it is observed that the research used a mixed methodology, which involved a technological and descriptive processing of experiences originating from semi-structured interviews. Finally, the conclusions propose that this initiative requires more research studies derived from this process, in addition, gender normative need to be integrated into the strategies and objectives of AR4D to ensure livelihoods and food security, particularly for women. | Thank you for your positive comment and for highlighting the contribution of our paper. |
| **Reviewer #2**  7. Is the manuscript technically sound, and do the data support the conclusions? The manuscript must describe a technically sound piece of scientific research with data that supports the conclusions. Experiments must have been conducted rigorously, with appropriate controls, replication, and sample sizes. The conclusions must be drawn appropriately based on the data presented.  Reviewer #2: Yes | Thank you. |
| 8. Has the statistical analysis been performed appropriately and rigorously?  Reviewer #2: Yes | Thank you. |
| 9. Have the authors made all data underlying the findings in their manuscript fully available? The PLOS Data policy requires authors to make all data underlying the findings described in their manuscript fully available without restriction, with rare exception (please refer to the Data Availability Statement in the manuscript PDF file). The data should be provided as part of the manuscript or its supporting information, or deposited to a public repository. For example, in addition to summary statistics, the data points behind means, medians and variance measures should be available. If there are restrictions on publicly sharing data—e.g. participant privacy or use of data from a third party—those must be specified.  Reviewer #2: Yes | Thank you. |
| 10. Is the manuscript presented in an intelligible fashion and written in standard English? PLOS ONE does not copyedit accepted manuscripts, so the language in submitted articles must be clear, correct, and unambiguous. Any typographical or grammatical errors should be corrected at revision, so please note any specific errors here.  Reviewer #2: Yes | Thank you. |
| 11. Review Comments to the Author.  When compared to the original manuscript, there is a significant improvement. However, the conclusion focused on the findings rather than how applicable they were to the labor market and the industry. Proofreading is also required to correct a few mistakes in the work. | Thank you. We have carefully proofread our manuscript.  We have also added a sub-section in ‘Discussion and Reflections’ titled ‘Opportunities for AR4D interventions’ (lines 580-627) where we highlight potential ways to apply our findings. Likewise, we have added a paragraph about this in the ‘Conclusions’ (lines 661-664). |
